# Differential DNA Methylation Correlates with Differential Expression of Angiogenic Factors in Human Heart Failure

**DOI:** 10.1371/journal.pone.0008564

**Published:** 2010-01-13

**Authors:** Mehregan Movassagh, Mun-Kit Choy, Martin Goddard, Martin R. Bennett, Thomas A. Down, Roger S.-Y. Foo

**Affiliations:** 1 Division of Cardiovascular Medicine, Addenbrooke's Centre for Clinical Investigation, University of Cambridge, Cambridge, United Kingdom; 2 Department of Histopathology, Papworth Hospital, Cambridge, United Kingdom; 3 Cancer Research UK Gurdon Institute, University of Cambridge, Cambridge, United Kingdom; Texas A&M University, United States of America

## Abstract

Epigenetic mechanisms such as microRNA and histone modification are crucially responsible for dysregulated gene expression in heart failure. In contrast, the role of DNA methylation, another well-characterized epigenetic mark, is unknown. In order to examine whether human cardiomyopathy of different etiologies are connected by a unifying pattern of DNA methylation pattern, we undertook profiling with ischaemic and idiopathic end-stage cardiomyopathic left ventricular (LV) explants from patients who had undergone cardiac transplantation compared to normal control. We performed a preliminary analysis using methylated-DNA immunoprecipitation-chip (MeDIP-chip), validated differential methylation loci by bisulfite-(BS) PCR and high throughput sequencing, and identified 3 angiogenesis-related genetic loci that were differentially methylated. Using quantitative RT-PCR, we found that the expression of these genes differed significantly between CM hearts and normal control (*p*<0.01). Moreover, for each individual LV tissue, differential methylation showed a predicted correlation to differential expression of the corresponding gene. Thus, differential DNA methylation exists in human cardiomyopathy. In this series of heterogenous cardiomyopathic LV explants, differential DNA methylation was found in at least 3 angiogenesis-related genes. While in other systems, changes in DNA methylation at specific genomic loci usually precede changes in the expression of corresponding genes, our current findings in cardiomyopathy merit further investigation to determine whether DNA methylation changes play a causative role in the progression of heart failure.

## Introduction

The pathogenesis of heart failure involves molecular mechanisms which are becoming better understood [1], [2] and studies in both experimental models and humans demonstrate the important relevance of dysregulated gene expression [1], [3]. Furthermore, transcriptomic analyses of human dilated cardiomyopathy show a consistent and distinct pattern of gene expression [4], and dysregulated expression of both coding and non-coding genes directly affects heart failure development and progression [3], [5].

Higher order mechanisms such as microRNA [5], [6] and histone modifications [7], [8] can alter gene expression control in cardiovascular disease, without involving any change in the underlying DNA sequence. The direct covalent modification of DNA cytosine nucleotides by methylation is another well-recognized epigenetic mechanism but its role in cardiovascular disease is unknown. DNA cytosine-methylation alters accessibility for transcription factor complexes at a local level and, like histone modifications, affects chromatin structure at regional and genome-wide levels. A well-characterised functional effect of DNA methylation is thus control of gene expression [9]. In this respect, hypomethylation of the 5′ promoter end of a gene correlates with increased expression of the gene [9], whereas hypermethylation within the body of a gene, is linked to increased gene expression [10], [11]. Although a direct molecular mechanism to explain these observations is still lacking, basic mechanisms by which DNA methyl-transferases and putative DNA de-methylase complexes function have recently been uncovered [12]–[14]. Complementary to this, one of the most important findings in the field of epigenetics in recent times is the discovery of DNA methylation variation between individuals at a genome-wide level [15]. Evidence indicates that this variation may be in part inherited [15] and in part, acquired [16], [17]. At least through its control of gene expression, DNA methylation variation may account for complex disease susceptibility or progression [15], [18].

For instance, Fraga *et al* [19] found that although monozygotic twins share a common genotype and DNA methylation was indistinguishable in younger twins, older twins exhibited remarkable methylation differences that correlated with a pattern of differential gene expression. Similarly, consistent with the notion of drifts in DNA methylation with increasing age, acquired variation in DNA methylation has been attributed to causes such as environmental, hormonal and stochastic events [16], [17], [20]. Differential DNA methylation, either through its influence on gene expression or other yet unknown mechanisms, could therefore explain differences in disease susceptibility or phenotypic discordance seen in monozygotic twin pairs in spite of their identical DNA sequences. In the wider population, differential DNA methylation may similarly contribute to the diversity of phenotypes, pathogenesis and progression of complex diseases. Apart from the strong association already identified between differential DNA methylation and cancer [21], there are now on-going efforts to investigate the link between DNA methylation variation and other complex diseases such as schizophrenia [22], diabetes [23] and inflammatory bowel disease [24].

Here, we tested the hypothesis that global DNA methylation profiles in human cardiac tissue differ between cardiomyopathy and normal control, and aimed to identify a subset of genomic loci whose differential methylation is correlated to differential expression of their corresponding genes.

## Results

### Differentially Methylated DNA Regions in Dilated Cardiomyopathy (CM-DMRs)

As a proof of concept that DNA methylation in a subset of genomic loci may connect end-stage cardiomyopathy with different etiologies, we set out initially to profile a series of heterogenous cardiomyopathic left ventricles and a single normal control (diseased: samples I, II, III; control: sample A; Table 1) using MeDIP-chip (Method summarised in Figure S1). We used the Nimblegen “CpG island and promoter” microarray chip (Roche Nimblegen, WI) which covers all annotated Human Refseq gene promoters (24,659) and CpG islands (28,226) as annotated on the UCSC genome browser.

**Table 1:** Details of patient LV samples.

LV sample	Code	Age	Details	Medications
Control	A	41	Asian	-
	B	47	RTA	-
	C	50	RTA	-
	D	41	RTA	-
	E	52	RTA	-
	F	50	Hypoxic brain damage secondary to drowning	-
End-stage cardiomyopathy	I	49	LV non-compaction, no history of coronary artery disease	Bumetanine, Amiodarone, Enalapril
	II	53	Idiopathic, no history of CAD	Aspirin. Bisoprolol, Frusemide, Spironolactone
	III	64	Ischaemic, diabetic	Clopidogrel, Ramipril, Digoxin, Spironolactone, Bumetanide, Gliclazide, Simvastatin
	IV	42	Idiopathic, no history of CAD	Perindopril, Carvedilol, Warfarin
	V	64	Idiopathic, no history of CAD	Frusemide, Spironolactone, Lisinopril, Amiodarone, Atorvastatin, Warfarin
	VI	61	Ischaemic	Clopidogrel, Perindopril, Nicorandil, Bisoprolol, Imdur, Spironolactone, Frusemide, Digoxin, Simvastatin
	VII	60	Ischaemic, diabetic	Aspirin, Clopidogrel, Ramipril, Bisoprolol, Frusemide, Spironolactone, Frusemide, Insulin
	VIII	58	Ischaemic	Aspirin, Ramipril, Carvedilol, Frusemide, Digoxin, Atorvastatin

RTA: road traffic accident; all LV samples were from Caucasian males (except A: Asian male).

CAD: coronary artery disease.

**Figure 1:**
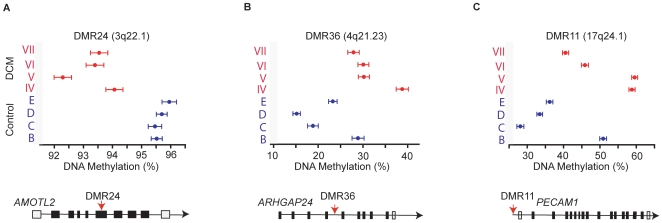
Differential DNA methylation profile for 3 candidate CM-DMR. (A) DMR24, (B) DMR36 and (C) DMR11. DNA methylation (%) was determined for a set of 8 LV samples (4 controls: B–E, 4 diseased (CM): IV–VII) by BS-PCR-sequencing as detailed in methods. *Lower panel*, DMR24 and DMR36 lie within the body of the genes: *AMOTL2* and *ARHGAP24*; whereas DMR11 lies in the 5′ regulatory region of *PECAM1*.

Based on a DMR (differentially methylated region) T-statistic>+3.0 and <−3.0, candidate regions (CM-DMRs) were identified using the validated algorithm-Bayesian tool that had been developed specifically for handling MeDIP data (BATMAN)[25]–[26] (Figures S1 and S2). Of the target CM-DMRs meeting these criteria, three candidates were identified by gene ontology analysis and GENECARDS to relate to angiogenesis (*AMOTL2*, *ARHGAP24* and *PECAM1*, Figure 1 and Figure S4). Next, we undertook bisulfite-(BS) PCR and massive parallel amplicon sequencing for these CM-DMRs using a second and larger set of LV samples (diseased: III, IV, V, VI; and controls: B, C, D, E; Table 1). Bisulfite treatment of gDNA converts unmethylated-cytosine nucleotides to uracil but leaves methylated-cytosine residues unaffected. This difference is then detected as a C/T nucleotide polymorphism at each CpG site by subsequent PCR and sequencing, providing the gold-standard high-resolution information about the methylation status of a DNA region. Amplicon sequences were matched to the reference Human Genome and DNA methylation (%) for each locus was determined (Figure 1).

### Differential Expression of Genes Related to CM-DMR

Although epigenetics may direct complex effects including changes in higher-order chromatin structure, DNA methylation differences are at least currently understood to control gene expression. At the local DNA level, hypomethylation in the 5′ or promoter region of a gene is associated with increased transcription; whereas hypermethylation of the body of a gene is associated with its active transcription [10], [11]. We therefore set out to determine expression of the target genes that we have identified.

Before quantifying target gene expression, we ascertained that our LV samples had at least 10-fold upregulated levels of *NPPA* mRNA compared to controls (Figure S3, *p* = 0.008). *NPPA* is upregulated in cardiomyopathy and represents the fetal gene program re-induction that is expected for myocardial disease [1]. Differential DNA methylation at the *NPPA* gene CpG island and promoter locus was however absent on MeDIP-chip (data not shown), indicating that *NPPA* gene expression may not be controlled by DNA methylation in this context or differential *NPPA* gene expression may correlate to differential methylation at a non-CpG island locus that was not present on the Nimblegen chip.

**Figure 2:**
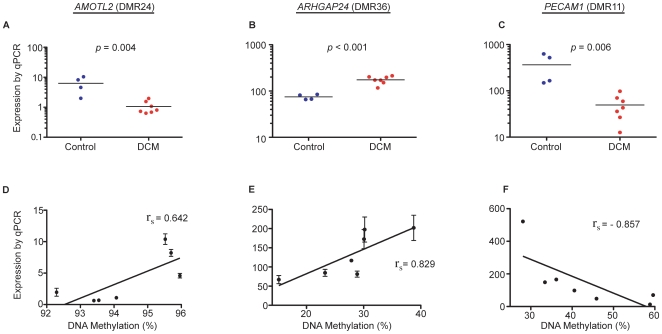
Differential DNA methylation for 3 CM-DMR correlates with differential gene expression. (A–C) Gene expression profiles for the gene corresponding to each DMR: *AMOTL2*, *ARHGAP24*, and *PECAM1*. Quantitative PCR was performed in a set of 11 LV samples (4 controls: C–F, 7 CM: II–VIII). QPCR experiments were performed in triplicate for each sample. ** p<0.05. (D–F) Correlation between gene expression and DNA methylation using Spearmans rank order correlation coefficient.

In contrast, qPCR for transcripts of the 3 genes that were associated with the 3 CM-DMRs that we had identified, showed statistically significant differential expression between control LV and CM LV (Figure 2; *p* = 0.004 for *AMOLT2*, *p*<0.001 for *ARHGAP24*, *p* = 0.006 for *PECAM1*). Consistent with the predicted effect of hypermethylation in the 5′ regulatory region of genes, hypermethylation in the 5′ region of *PECAM1* in dilated hearts (DMR11, Figure 1C) correlated with decreased expression of *PECAM1* (Figure 2C). Similarly consistent with the predicted effect of gene body methylation, hypomethylation within the gene body of *AMOTL2* (DMR24, Figure 1A) correlated with reduced expression of *AMOTL2* (Figure 2A); and hypermethylation within the gene body of *ARHGAP24* (DMR36, Figure 1B) correlated with increased expression of *ARHGAP24* (Figure 2B).

We further analysed the correlation between gene expression and differential methylation in each individual LV tissue directly and independently of disease, and found as predicted, that 5′ region methylation correlated inversely to gene expression (DMR11 in Figure 2F). Similarly for DMR24 and DMR36, a positive correlation existed between gene body methylation and gene expression (Figure 2D and E). Importantly, although differential gene expression patterns were opposite for *AMOTL2* and *ARHGAP24* in dilated hearts (decreased and increased expression respectively), the correlation between methylation and gene expression was positive in both. Moreover, individual LV samples with the highest or lowest expression of each of these genes mapped to the predicted methylation state in their correlation between methylation and expression independently of disease state.

## Discussion

In eukaryotes, DNA methylation occurs by the addition of a methyl group to the carbon 5′ position of the nucleotide cytosine ring, and cytosine methylation in mammals, is found most commonly in the context of the sequence 5′-CG-3′, which is also referred to as a CpG dinucleotide. In the mammalian genome, an estimated 70% of all CpGs are methylated [27]. Unmethylated CpG on the other hand are largely grouped in clusters called “CpG islands” in the 5′ regulatory region of many genes. The frequency of CpG dinucleotides in “CpG islands” is higher than is found in other DNA regions. Notably, differential methylation of CpG islands is part of the epigenetic variation found in humans [18], [27].

Consistent with previous observations correlating gene expression with DNA methylation [9]–[11], we have found that hypermethylation within the 5′ region of the *PECAM1* gene correlated with its reduced expression in different cardiac samples. Hypermethylation within the body of the *ARHGAP24* gene correlated with its increased expression; and hypomethylation within the body of the *AMOTL2* gene correlated with its decreased expression. Although our study was not designed to address the question of causality, current evidence suggests that locus-specific DNA methylation either permissively or necessarily controls gene-specific expression [16], [17], [21], [27]. This may occur by the binding of methylated DNA binding domain (MBD) proteins and polycomb group proteins which displaces the transcription machinery and thereby maintaining epigenetic silencing of transcriptional activity [9]. The hypermethylation mark may in some cases, be laid down subsequent to transcriptional down-regulation of a gene [28], but experiments using the DNA methyl-transferase inhibitor (5′-deoxyazacytidine) show that de-methylation of a specific gene promoter can re-establish gene expression [21], [29]. Similarly the interaction between histone modification and DNA methylation in regulating gene expression is currently unclear, and may indeed be locus specific. At least in some cases, histone deacetylase inhibitors such as Trichostatin A is also required with de-methylating agents in order to restore gene expression in an otherwise densely methylated gene locus [30]. The interaction between histone de-acetylation and DNA methylation in myocardial disease will be particularly important to understand since the critical role of histone deacetylases (HDAC) in cardiac hypertrophy and heart failure has already been established [8], [31].

This is the first demonstration that differential DNA methylation exists between human end-stage cardiomyopathic hearts and normal controls. The utility of the MeDIP-chip dataset may be limited because only a single control heart was used, but we have also used a second series of hearts and verified using the second methodology of bisulphite sequencing, that differential methylation exists in heart failure. Moreover despite the heterogeneity of our samples, we have found that for at least 3 genomic loci, differential methylation corresponds to differential gene expression. This was anticipated since several disease processes and molecular pathways such as apoptosis, dyregulated calcium signalling, decompensated contractility, G-coupled protein receptor down-regulation, and maladaptive angiogenesis also connect cardiomyopathy of disparate etiologies [2]. Dysregulated gene expression and differential DNA methylation of angiogenic factors may indeed connect heart failure with different etiologies, in the same manner that raised levels of *NPPA* and the re-induction of a fetal gene programme marks heart failure irregardless of its inciting cause [2]. As with cDNA microarray experiments utilizing whole heart tissue with mixed cell populations, our findings may also reflect a change in the predominance of a particular cell type in each tissue sample. We have however not detected any expression pattern change in gene families that are characteristically specific to non-myocyte cell types such as fibroblasts (data not shown).

At a broad genomic level, 3-dimensional intra-chromosomal and inter-chromosomal DNA-DNA interactions may partition the genome into active and inactive domains [32]. These interactions characteristically involve DNA regulatory elements. Protein complexes comprising of DNA-binding proteins bound to these regulatory elements may hold together DNA conformations to form chromatin centres of active transcription or transcriptional factories. By altering access to DNA-binding proteins, different DNA methylation states of these DNA regulatory elements in disease versus control may therefore be responsible for important changes in 3-D conformations and hene, gene expression [33]. Although in this study, we have examined the effect of DNA methylation on local proximal gene expression, instead of a linear series of genes and promoters, the 3-D model of transcriptional regulatory networks [32] suggests that altered DNA methylation may influence the expression of genes in sites that are distal or distant [33]. Nevertheless for the control of proximal gene expression, others have previously reported that a 6% methylation difference within the *RASSF1* gene comparing between methylation profiles of the cerebellum and cerebrum corresponded with a 2-fold difference in *RASSF1* gene expression in these 2 different parts of the brain [22]. More recently Barres *et al* showed that 2–5% methylation difference in the *PGC1A* promoter related to a 3.5 fold difference in PGC1α gene expression in the *vastus lateralis* muscle of patients with type II diabetes [23]. In breast cancer, 8% methylation difference was associated with a 1.5 to 3.5 fold expression difference of the *ATM* gene [34]; and comparing between children conceived *in vitro* or *in vivo*, 7% and 9.7% methylation difference in the *COPG2* and *CEBPA* genes related to 2.05 and 1.77 fold changes in gene expression respectively [35]. Using the BS-PCR-sequencing strategy we have found here that differential methylation between control and CM ranged up to 32% (Figure S1 and data not shown). More importantly, we have demonstrated the biological significance for this range of differential methylation in 3 DMRs. In the case of DMR24/*AMOTL2*, 2–3% differential methylation between diseased hearts and controls correlated with a 2.5-fold decrease in *AMOTL2* gene expression. For DMR36/*ARHGAP24*, a 3–5% difference in methylation corresponded to a 2.5-fold increase in gene expression.

Functionally, *AMOTL2* belongs to the angiomotin family which mediates inhibition of endothelial cell migration and tube formation by binding to angiostatin [36]. *ARHGAP* genes encode RhoGAP family proteins, and using subtraction-hybridization in endothelial cells undergoing capillary-tube formation, *ARHGAP24* (also known as *p73RhoGAP*) was also found to regulate capillary-tube formation [37]. *ARHGAP24* expression was up-regulated in an angiogenic milieu but unchanged under non-angiogenic conditions [38]. The role of *PECAM1* (or CD31) and regulation of its expression in angiogenesis have also long been investigated [38], [39]. Our current findings implicating the differential expression of these 3 genes in end-stage heart failure may reflect adaptive or maladaptive angiogenic processes in disease pathogenesis, and will require further direct investigation. Even so, a transcriptomic analysis that was performed recently using endomyocardial biopsies from patients with new onset heart failure revealed that disease prognosis may be predicted based on the expression profile of a series of genes included genes of angiogenic factors, one of which was *ARHGAP26* [4]. Moreover, the important role of angiogenesis has been demonstrated before, both in human ischaemic heart disease [40] and experimental models of non-ischaemic related heart failure [41].

Our results show that differential DNA methylation occurs in human end-stage cardiomyopathy. Gene expression is dysregulated in heart failure and a subset of this connecting end-stage disease with different etiologies may be explained by differential DNA methylation together with other epigenetic mechanisms such as histone de-acetylation. As these epigenetic mechanisms may be altered by the environment and diet, differential DNA methylation may be responsible for integrating environmental/dietary signals and inherited traits to influence heart failure pathogenesis and progression. Larger studies will be needed to identify other differentially methylated genomic loci that bear a statistically significant association to heart failure. Moreover recent evidence suggests that future analysis should also include DNA regions outside of CpG islands because at least in human colon cancer, significant methylation variation was found in sequences >2 kb away from promoters and CpG islands (termed “CpG island shores”) [42]. Unravelling these additional complex layere of gene expression control will improve therapeutic options and alter patient management for this complex disease.

## Materials and Methods

### Ethics Statement

Human myocardium was collected by a protocol approved by the Papworth (Cambridge) Hospital Tissue Bank review board and the Cambridgeshire Research Ethics Committee (UK). Written consent was obtained from every individual according to the Papworth Tissue Bank protocol.

### Human Left Ventricular Myocardium

Left ventricular (LV) tissue was obtained from male patients undergoing cardiac transplant for end-stage heart failure. Normal non-donor suitable human LV tissue was from healthy male individuals involved in road traffic accidents. At the time of transplantation or donor harvest, whole hearts were removed after preservation and transported in cold cardioplegic solution (cardioplegia formula and Hartmann's solution) similar to the procedure described before at Imperial College, London [43]. Following analysis by a cardiovascular pathologist (M.G.), left ventricular segments were cut and stored immediately in RNAlater (Ambion, Austin, Tx). Individual patient details are listed in Table 1. Integrity of genomic DNA (gDNA) and RNA isolated from each tissue was verified by Nanodrop (Thermo Scientific, Wilmington, DE), QIAxcel system (Qiagen, Crawley, West Sussex, UK) (for DNA) and 2100 Bioanalyzer (Agilent Technologies, Stockport, Cheshire, UK) (for RNA).

### Genomic DNA Isolation

gDNA was isolated from LV samples using Genomic DNA G100 Tips (Qiagen, Crawley, UK). Samples were homogenised using a hand-held homogenizer, treated with 200 µg/ml RNase A (Qiagen) for 15 min at room temperature and thereafter digested with 1 mg/ml Proteinase K (Roche Diagnostics, Burgess Hill, UK) overnight.

### Methylated-Cytosine DNA Immunoprecipitation–Microarray Chip (MeDIP-Chip)

According to protocols previously described (26–28), at least 25 µg of gDNA sample was diluted in TE buffer (10 mM Tris-HCl, pH 7.5, 1 mM EDTA) and sheared to between 100–800 bp fragments using Bioruptor (Diagenode, Belgium). 4 µg of each sample was saved as INPUT and the rest heated to 95°C for 10 min and immediately placed on ice. Immunoprecipitated was performed using 2.5 µg of α-5′methyl-cytosine antibody per µg of sheared gDNA in IP buffer (20 mM Na-Phosphate, pH 7.0, 1 M NaCl, 2% Triton-X100). Samples were rotated overnight at 4°C and 10 µl of 50% Protein-A Agarose slurry (pre-washed in 0.1% BSA-PBS and equilibrated in IP buffer) was subsequently added per µg of DNA. Samples were rotated for further 2.5 hr and washed 3 times with IP buffer before elution using 250 µl lysis buffer (1 M Tris-HCl, pH 8.0, 0.5 M EDTA, 10% SDS, 280 µg/ml Proteinase K) and incubation for 2 hr at 55°C. MeDIP was purified and precipitated using phenol and chloroform:isoamyl alcohol. The extent of methylated DNA enrichment in our MeDIP samples was verified by qPCR for the normally methylated target region of *OXT* (Figure S1B and Figure S4). We also verified the expected depletion of the unmethylated target region of *UBE2B*.

Four-µg of INPUT and MeDIP for each sample were labelled with Cy3 and Cy5, respectively and co-hybridised to the Nimblegen “CpG island and promoter” microarray chip (Nimblegen, WI). This Nimblegen array chip comprises of 385,000 isothermal probes of between 50–75 mer length with a median probe spacing of 101 bp, and is based on the HG18 human genome assembly. These probes cover all reported Human Refseq gene promoters (24,659) that ranged from −800 bp to +200 bp relative to transcription start sites (TSS) and all reported CpG islands (28,226) annotated on the UCSC genome browser.

### Bisulfite Treatment of gDNA, PCR and Massive Parallel Amplicon Sequencing

Bisulfite (BS) conversion of gDNA was performed using the EZ DNA Methylation-Gold kit (Zymo Research, Orange, CA) according to manufacturer's protocol. PCR was performed using BS-treated gDNA samples as template, and BS-specific primers that were designed at a minimum length of 20 bp against selected DMRs using the MethPrimer program. (http://www.urogene.org/methprimer/index1.html). Composite primers were designed by incorporating 19 bp Roche FLX Primer-A and Primer-B specific sequences to the 5′-ends of Forward and Reverse primers respectively.

Primers were tested to confirm the amplification of BS-treated gDNA only and individual PCR products were run on 2% agarose gels to verify product size (data not shown). Concentration of PCR amplicons was determined using the QIAxcel system and amplicons from each LV sample were pooled at equimolar concentrations and sequenced on individual lanes in a Next Generation 454 FLX sequencing machine (Roche). We obtained between a 4.4–7.3 million bp reads per sample per lane of FLX sequencing which corresponds to an average number of 24,000 reads of 250 bp average product size per lane. This in turn corresponded to an average depth of about 1,100 reads per individual PCR product.

Amplicon sequence reads corresponding to the BS-PCR products were aligned to the Human Reference Genome, and the extent of methylation (DNA methylation%) was determined by comparing the total number of Cs (methylated) to Ts (unmethylated) for each CpG site in a single DMR.

### RNA Extraction, cDNA Synthesis and Expression Quantitative PCR

At least 30 mg of frozen LV sample was thawed in 1 ml of TRI-reagent (Sigma-Aldrich, St Louis, MO) and homogenised for 3 times 20 sec bursts, using the Lysing Matrix (QBiogene, Cambridge, UK) in a FastPrep machine (FP120, QBiogene). Thereafter, beads were centrifuged at 3000 rpm for 3 min, supernatant transferred to a clean Eppendorf and RNA extraction was performed according to manufacturer's protocol. Twenty-µl of cDNA was synthesised from 1 µg of Total RNA, using a mixture of both oligo-dT and random hexamers and the “Superscript-III first strand cDNA synthesis kit” (Invitrogen, Paisley, UK). Integrity of RNA for all samples was checked using the 2100 Bioanalyzer (Agilent Technologies). Quantitative real-time PCR for house keeping genes was initially performed using 4 µl of 1∶20 pre-diluted cDNA in a 20 µl reaction and Taqman Gene Expression Assays specific for 18S, GAPDH, RPLPO and TBP. Using geNorm (http://medgen.ugent.be/~jvdesomp/genorm/#PrimerDesign) we determined that of these housekeeping genes, RPLPO was most stable for both control and diseased samples. Quantitative PCR performed for target genes using validated Taqman Gene Expression Assay primers (Applied Biosystems, Foster City, CA) was therefore normalised against RPLPO. Quantitative PCR for both target genes and RPLPO were performed at least in triplicate on the same diluted cDNA samples.

### Statistical Analysis

Analysis for MeDIP-chip was performed using the BATMAN algorithm as previously described (27). Analysis for quantitative PCR was performed using the non-parametric Mann-Whitney t-test and two-tailed p values were used to determine statistical significance. Analysis for the association between DNA methylation and gene expression was performed using Spearmans rank order correlation coefficient.

## Supporting Information

Figure S1 Methodology (A) Work flow for MeDIP-chip and BS-PCR-sequencing using different sets of LV samples. (B) Enrichment for methylated gene, OXT, and non-enrichment of unmethylated gene, UBE2B, demonstrated by qPCR validates the effectiveness of MeDIP.
(1.28 MB TIF)

Figure S2 Global differential DNA methylation in end-stage dilated cardiomyopathy. BATMAN analysis of MeDIP-chip for one control LV vs. average of 3 LV from end-stage cardiomyopathy hearts (CM). Heatmap represents BATMAN DMR T-statistic score.
(4.91 MB TIF)

Figure S3 Quantitative PCR for NPPA showing >10-fold upregulation in CM samples compared to control.
(0.20 MB TIF)

Figure S4 Examples for 3 CM-DMRs that were identified by MeDIP-chip in relation to an integrated resource of tissue-DMRs in other normal somatic and germ-line tissues (ref. 28).
(4.95 MB TIF)
