# Supplementary figures and images for: Differential DNA Methylation Correlates with Differential Expression of Angiogenic Factors in Human Heart Failure
Source: PLoS One. 2010 Jan 13;5(1):e8564. doi: 10.1371/journal.pone.0008564 (PMC2797324; doi:10.1371/journal.pone.0008564)

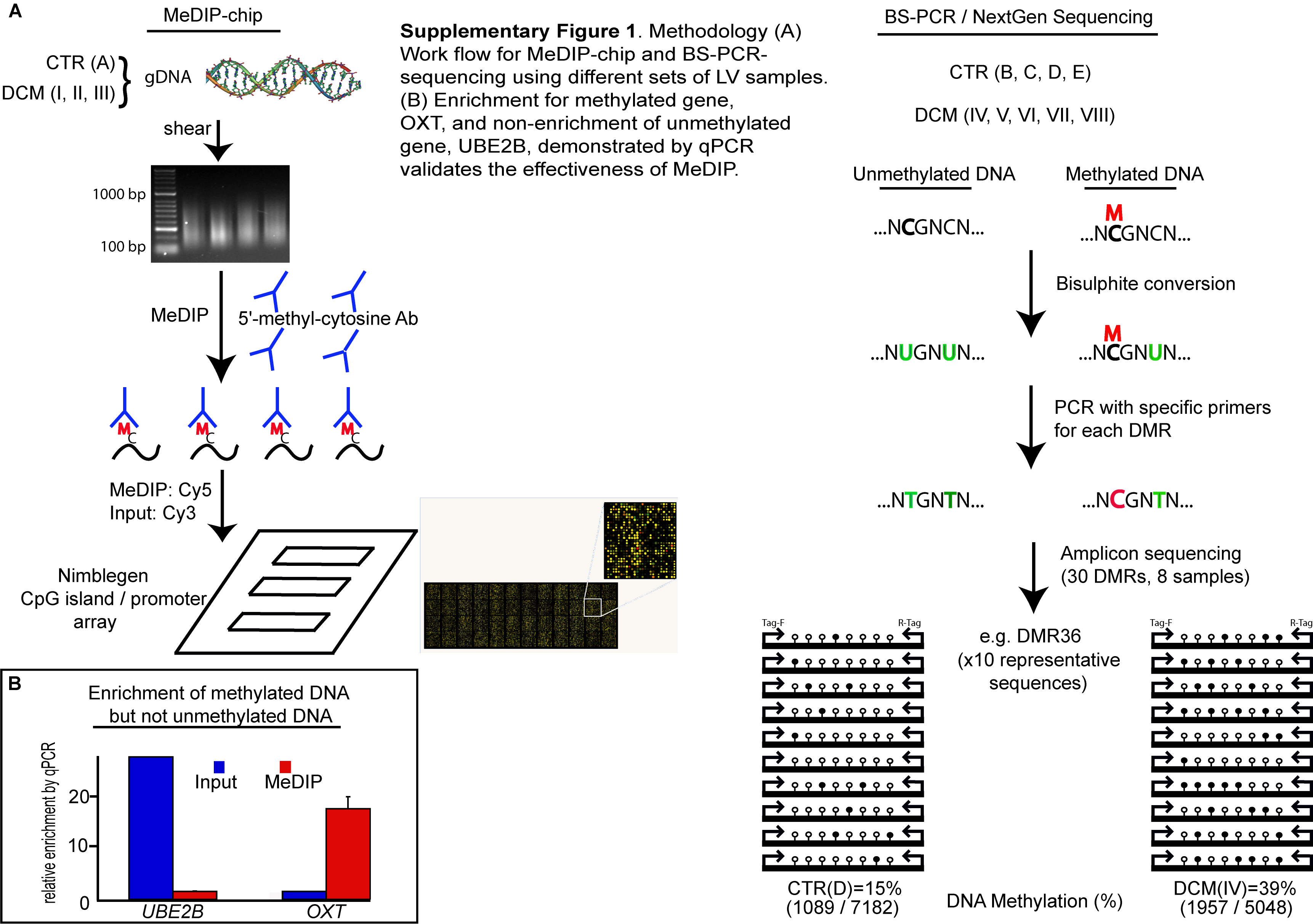

Supplement: Figure S1 — Methodology (A) Work flow for MeDIP-chip and BS-PCR-sequencing using different sets of LV samples. (B) Enrichment for methylated gene, OXT, and non-enrichment of unmethylated gene, UBE2B, demonstrated by qPCR validates the effectiveness of MeDIP. (1.28 MB TIF) [file pone.0008564.s001.tif]

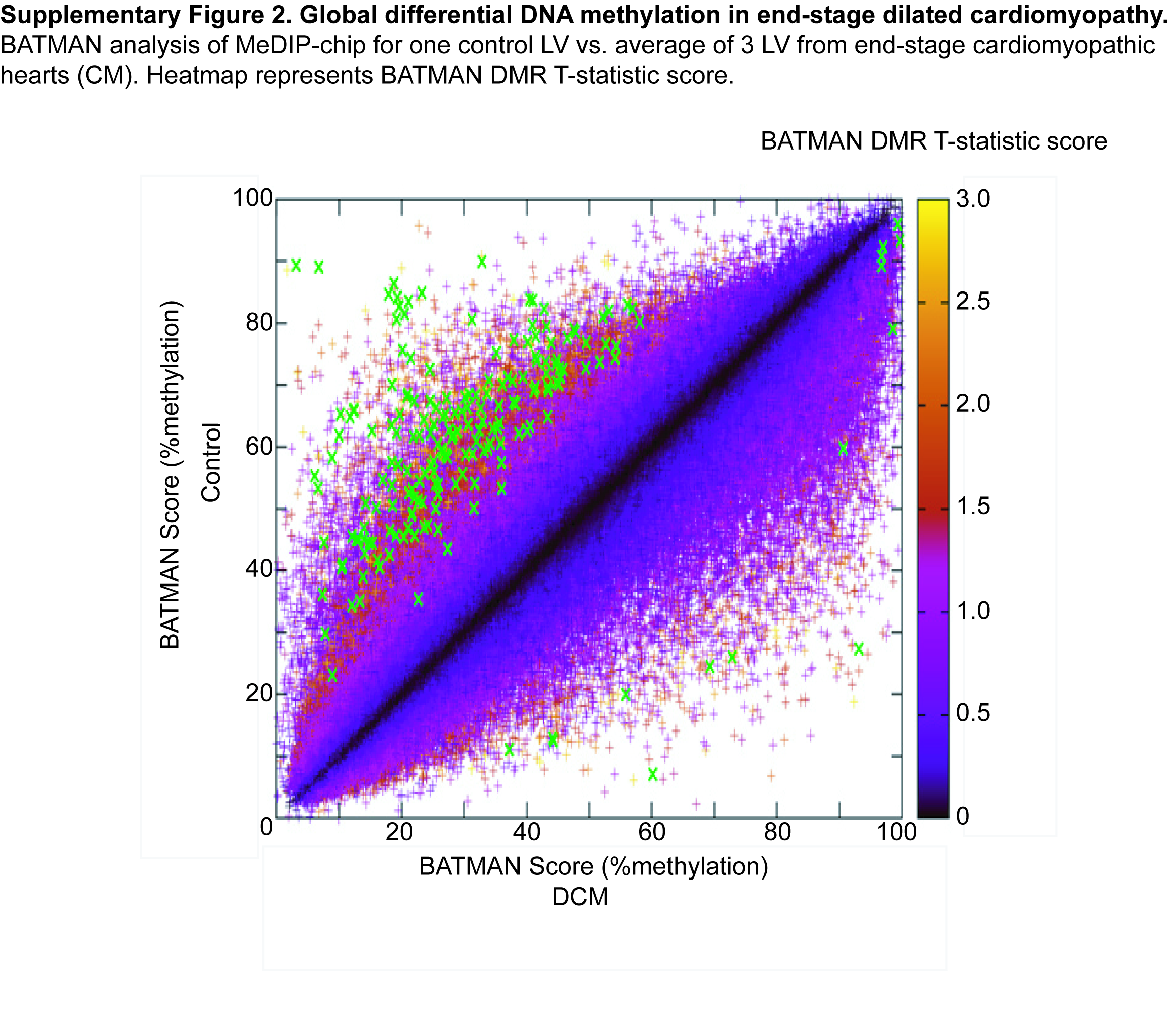

Supplement: Figure S2 — Global differential DNA methylation in end-stage dilated cardiomyopathy. BATMAN analysis of MeDIP-chip for one control LV vs. average of 3 LV from end-stage cardiomyopathy hearts (CM). Heatmap represents BATMAN DMR T-statistic score. (4.91 MB TIF) [file pone.0008564.s002.tif]

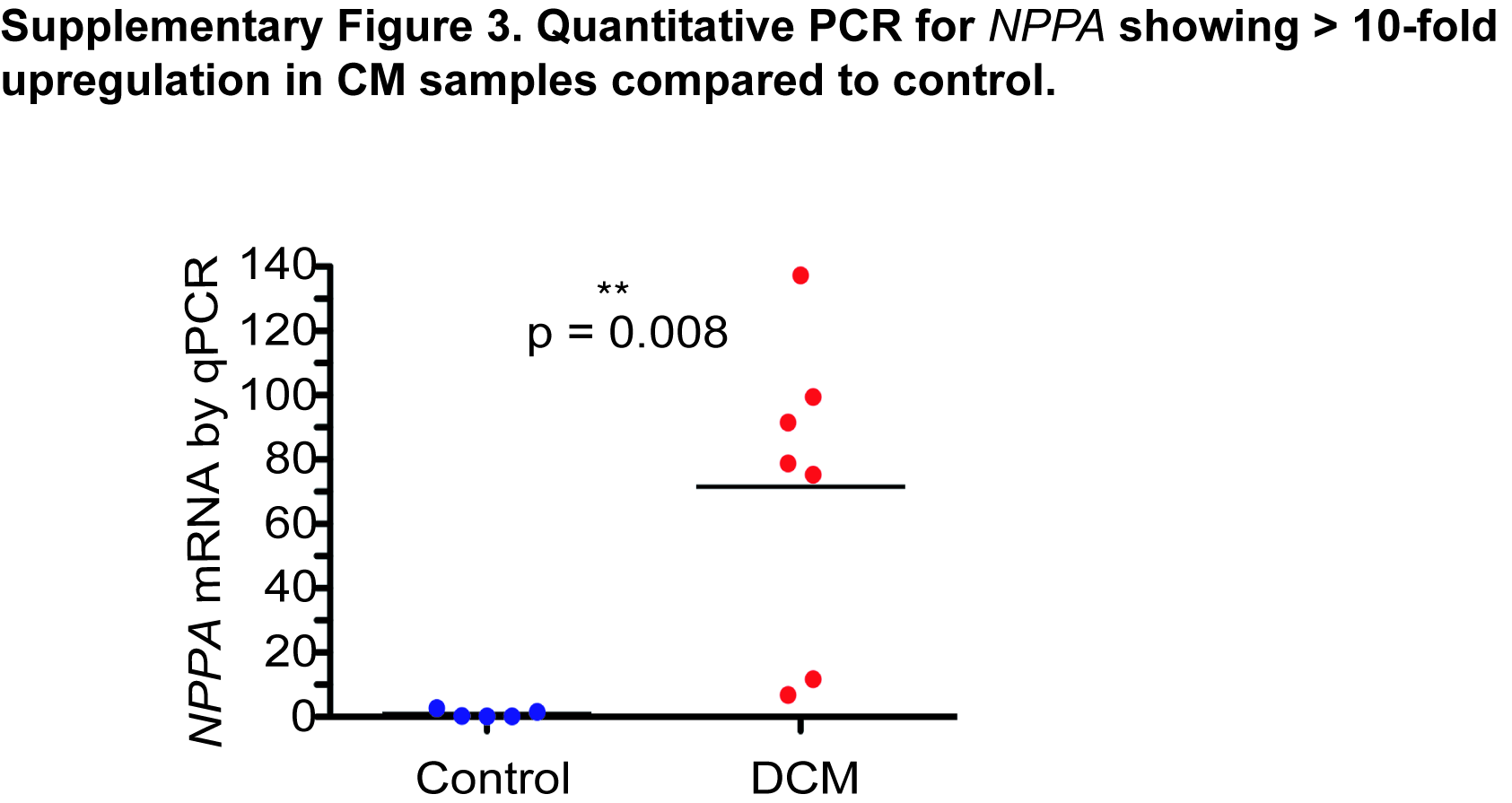

Supplement: Figure S3 — Quantitative PCR for NPPA showing >10-fold upregulation in CM samples compared to control. (0.20 MB TIF) [file pone.0008564.s003.tif]

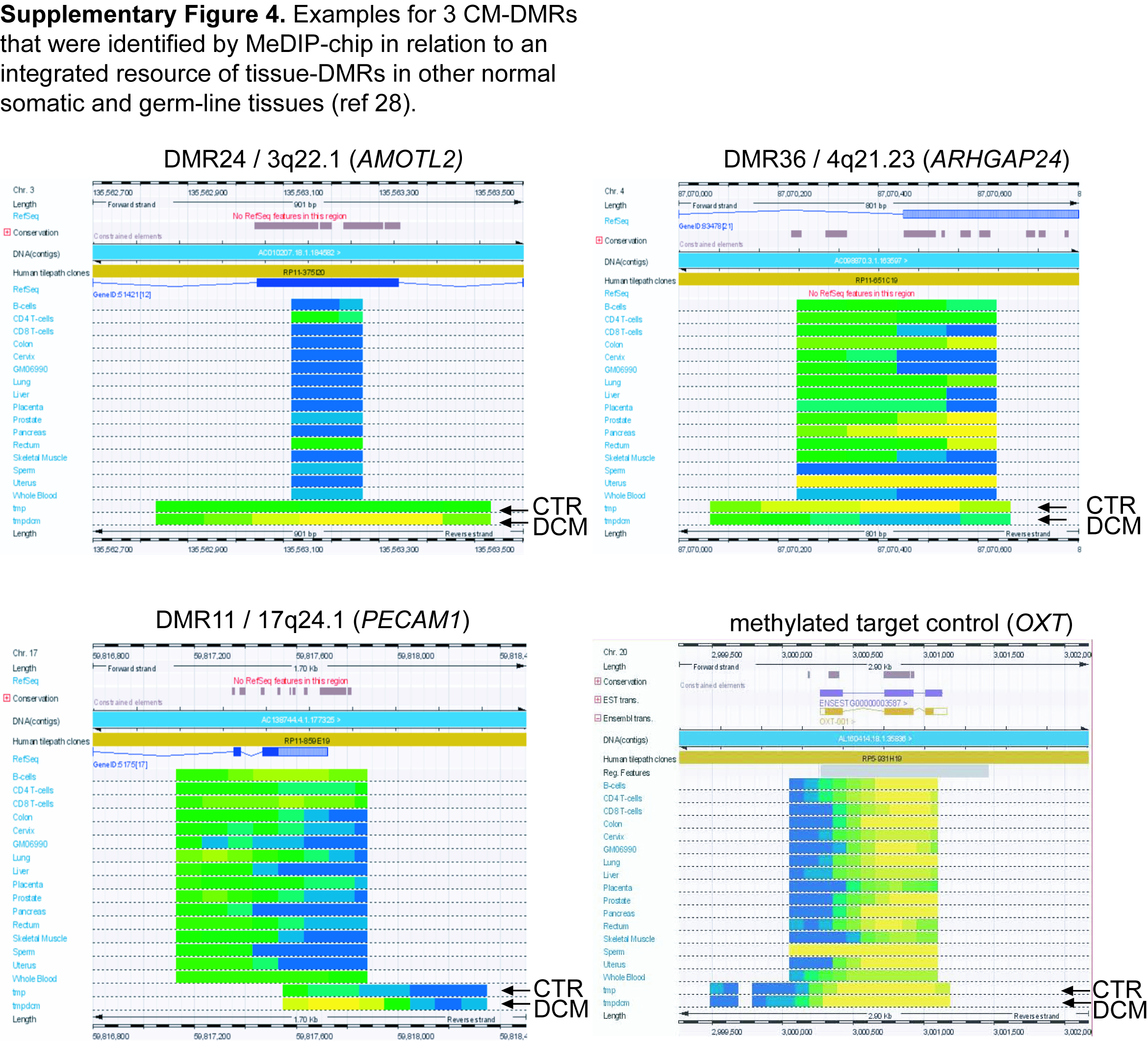

Supplement: Figure S4 — Examples for 3 CM-DMRs that were identified by MeDIP-chip in relation to an integrated resource of tissue-DMRs in other normal somatic and germ-line tissues (ref. 28). (4.95 MB TIF) [file pone.0008564.s004.tif]
